# Innovations on the horizon: teledentistry, artificial intelligence, and hybrid models to improve oral health for vulnerable communities

**DOI:** 10.3389/froh.2025.1649715

**Published:** 2025-08-11

**Authors:** Sergiu Drafta, Andrei Macris, Alexandru E. Petre

**Affiliations:** Prosthetic Department, Faculty of Stomatology, “Carol Davila” University of Medicine and Pharmacy from Bucharest, Bucharest, Romania

**Keywords:** teledentistry, artificial intelligence, dental care, oral health, dental diagnostic

## Abstract

**Objectives:**

Vulnerable populations experience substantial challenges accessing dental care, leading to disproportionate oral health burdens. Digital solutions, such as teledentistry and artificial intelligence, offer new opportunities to improve access, quality and effectiveness of the care.

**Methods:**

This paper presents a narrative literature review incorporating findings from peer-reviewed journals, systematic reviews, and case studies retrieved from PubMed, Scopus, and Web of Science (2013–2024). Evidence was also synthesized using the artificial intelligence tool Elicit to include high-quality data on teledentistry, artificial intelligence and hybrid dental care models, focusing on their implementation and effectiveness. Studies were selected to address relevance for underserved populations and digital innovation in dental care delivery.

**Results:**

Teledentistry modalities, including e-consults and virtual dental homes, have demonstarte enhanced access and delivery of preventative care. Artificial intelligence tool provide accurate diagnoses, predict treatment needs, and streamline workflows. Hybrid models combining virtual screening with in-person treatment bridge care gaps and enhance continuity and efficiency, particularly for underserved populations.

**Conclusions:**

The integration of teledentistry, artificial intelligence and hybrid dental practice models offers a transformative approach to improving oral health care acces and equity. However, success will require investment, support and collaboration to scale these innovations effectively.

## 1 Introduction

Access to quality oral health care remains a persistent challenge, particularly for vulnerable groups such as those living in rural areas (1), elderly (2–4), individuals with disabilities, and low-income populations (5). These groups often face difficulties accessing health care, high costs, and feelings of alienation, resulting inpronounced oral health disparities (6–8).

New technological tools have emerged to address these challenges. Teledentistry, a form of telemedicine that uses telecommunications to deliver dental care (2, 9–11) has developed rapidly. It includes synchronous e-consultation, asynchronous store-and-forward models, and virtual dental homes (12). These models enhance accessibility, reduce treatment delays, and lower care costs (4, 13, 14). However, challenges include the lack of physical examination and reduced face to face contact (14). Although 84% of healthcare workers find teledentistry convenient and easy to learn, 92% reported it to be time-consuming (15).

Artificial intelligence (AI) is useful to overcome these limitations by improving diagnostic accuracy through sophisticated imaging analysis and by enabling predictive, data-driven treatment planning (16, 17). AI can facilitate clinical decision-making and enhance workflow efficiency through quick processing of large volumes of data.

Hybrid dental care models combine the benefits of digital care and physical care. For example, AI-enabled mobile dental units integrated with teledentistry tools (18, 19) provide on-site screening and diagnosis, with in-clinic treatment if required. This hybrid approach improves care continuity and convenience.

Research demonstrates the high effectiveness of these hybrid dental care models. Deep learning-based diagnostics have achieved accuracies of 97% for detecting stains, 85% for caries, 83% for calculus (20), 88% for gingivitis, and 95% for identifying alveolar bone loss (21). Intraoral cameras and video consultations (19) represents a new frontier for teledentistry, expanding care to elderly and rural patients with satisfaction and reduced travel costs. Smartphone-based imaging (22) combined with telehealth software has shown a diagnostic sensitivity of 95% and specificity of 84% for oral lesions, comparable to face-to-face examinations (23).

Teledentistry has also been explored in various aspects of pediatric oral health, including general consultations and specialized treatments, particularly in underserved or rural settings. Examples include teledentistry for children aged 6–10 years in rural areas (15), pediatric dental consultations for children aged 3–13 years (24), school-based teledentistry programs for rural children (1), and orthodontic care for socially disadvantaged children (25, 26).

**Figure 1:**
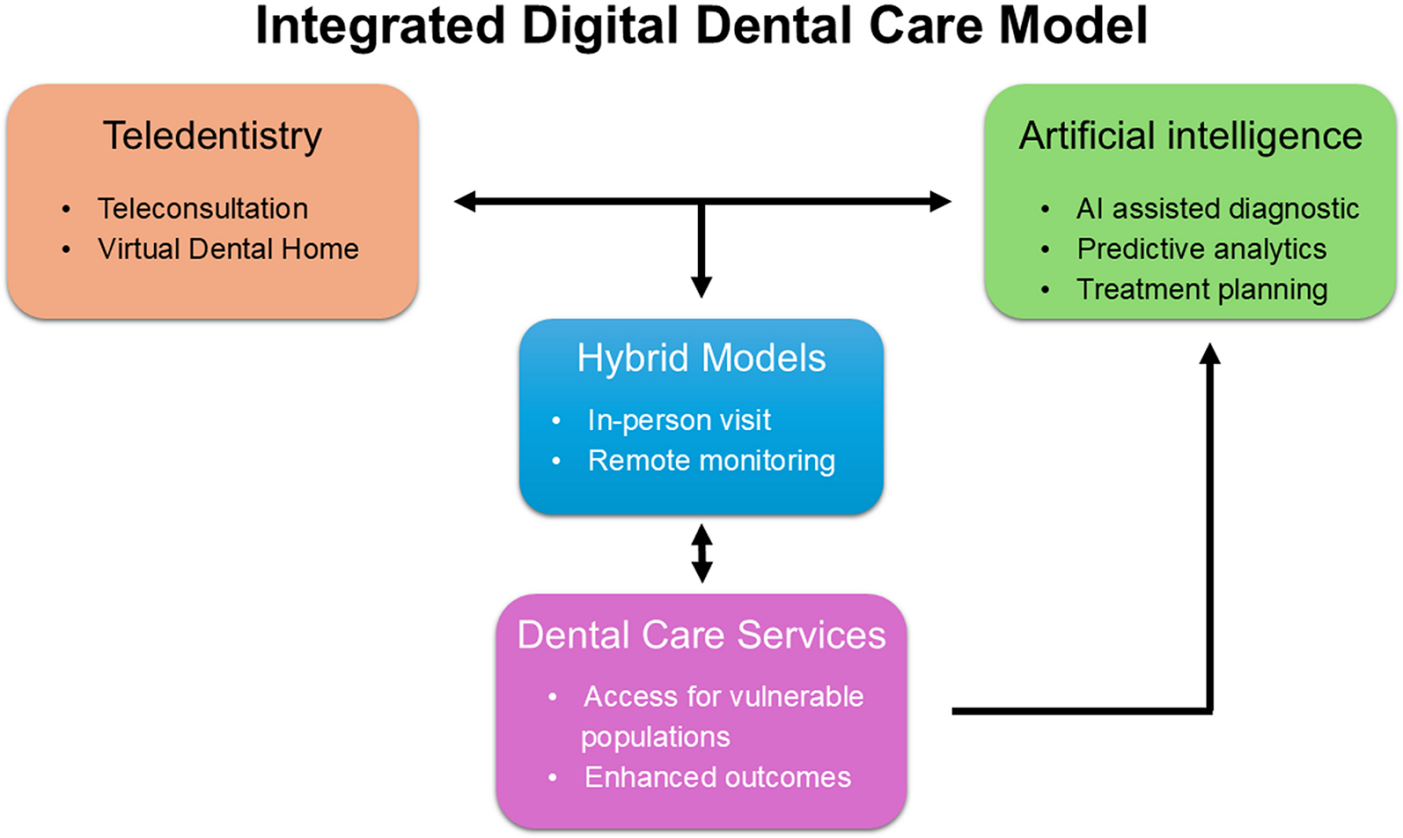
Workflow of an integrated digital dental care. A conceptual model showing the interaction between teledentistry, AI tools, and hybrid services delivery, aimed to impoving access and continuity of care for vulnerable populations.

This article examines how these synergistic innovations—teledentistry, AI, and hybrid dental care models—can enhance access, diagnosis accuracy, and patient experience (Figure 1). It draws on the latest research and real-world evidence, rather than relying solely on a limited set of project management tools. Figure 1 outlines the conceptual integration between AI tools and digital workflows.

The present study utilized AI tools and AI-driven web-platforms for searching, filtering and summarizing existing publications, including Elicit (Elicit Research, PBC, Covina, California, USA). Case studies analyzed through Elicit-filtered results include Virtual Dental Home (VDH) at the University of the Pacific (12), Apple Tree Dental (Apple Tree Dental EIN, New Brighton, Minnesota, USA), Overjet (Overjet Inc., San Mateo, California, USA), Pearl (HELLO PEARL Ltd., St Albans, United Kingdom), VideaHealth (16), DentalMonitoring (DentalMonitoring SAS, Paris, France), and Previsoft (Fojal SA, Groupe LEFEBVRE SARRUT, Ile-de-France, Paris, France). Related AI-powered tools, such as LogyAI (LOGY AI HEALTH INC., Wilmington, Delaware, USA), a newer AI software application, and Diagnocat (DGNCT LLC, Miami, Florida, USA), an established AI-based application, improve accuracy, reduce patient travel time, and enhance healthcare worker coordination.

## 2 Materials and methods

This narrative review synthesizes evidence from peer-reviewed journal articles, systematic reviews, case reports, and digital health implementation research related to teledentistry, AI, and hybrid dental care models. Literature searches were conducted in PubMed, Scopus, and Web of Science using a combination of keywords, including “teledentistry,” “AI in dentistry”, “virtual dental home”, “oral health technology”, and “hybrid dental models”. The review cover the literature published between 2013 and 2024.

In addition to manual searches, publications were identified and summarized using large language models available on the Elicit AI platform (Elicit Research, PBC, California, USA). This AI platform, which uses large language models, was employed in the initial phase of the literature search to identify potentially relevant articles based on keyword queries and structured prompts. While Elicit offers efficiency in narrowing down large sets of publications, its outputs depend on the current state of its underlying models, which may change over time, affecting reproducibility. Moreover, the selection process lacks full algorithmic transparency, and results may vary if queries are repeated at later dates. These limitations were addressed by manual verification at all critical decision points. The search tool retrieved and filtered 499 articles based on the following eligibility criteria:

- Focus on at-risk populations (e.g., rural, low-income, elderly, disabled, underserved children);
- Teledentistry or hybrid (digital or face-to-face) dental service delivery models;
- Development and application of AI-based dental diagnostic or screening tools;
- Primary research designs (e.g., randomized controlled trials, quasi-experimental studies, cohort studies, or systematic reviews/meta-analyses);
- Inclusion of quantifiable oral health or access outcomes;
- Emphasis on operationalizing non-clinical technologies rather than technological or provider development;
- Sample sizes of at least 10 patients providing original research data.

All inclusion criteria were also applied globally during the screening process. Titles and abstracts were screened, and a full-text reviews were conducted for studies meeting the inclusion criteria.

The Elicit platform was used to extract data on:

- Study type (e.g., pilot/feasibility, intervention, or comparative);
- Study location, population, age group, and the inclusion criteria;
- Teledentistry technology and delivery modality (e.g., intraoral camera use, real-time consultation, or hybrid integration);
- Intervention characteristics, including service level (screening, diagnosis, treatment, or education), and operator role;
- Key outcomes, such as diagnostic accuracy, access metrics, patient satisfaction, and *p*-values when available;
- Barriers and facilitators, implementation challenges, successful strategies, and participant/provider feedback.

**Figure 2:**
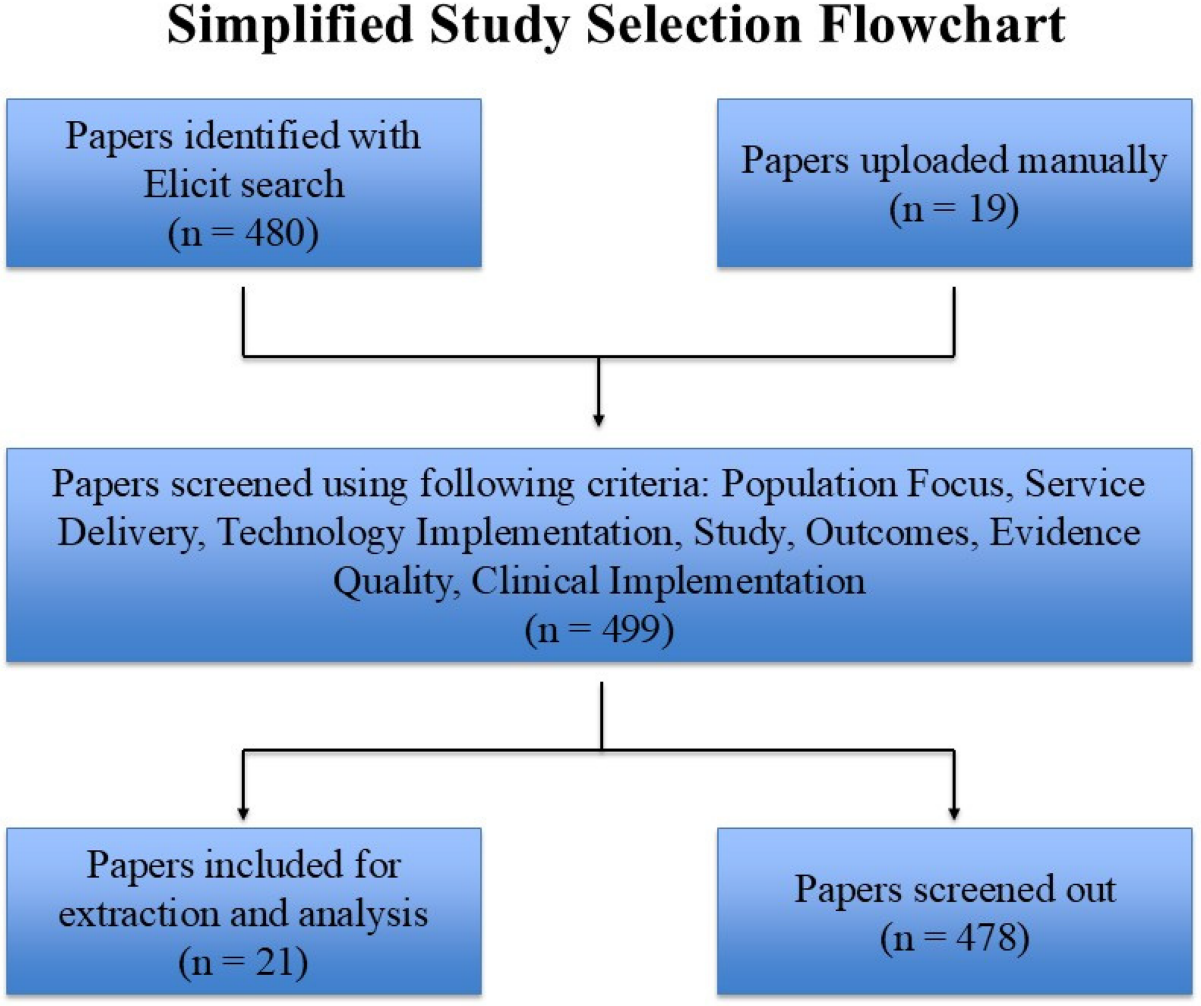
Flowchart illustrates the process of selecting studies for inclusion in the review, starting with papers identified through an elicit search and manually uploaded papers, followed by screening based on specific criteria.

A simplified flowchart illustrates the overall study selection process, providing visual clarity regarding inclusion steps (Figure 2).

This structured extraction approach enabled robust thematic synthesis and triangulation with manually reviewed studies. Case studies were drawn from established programs, such as the Virtual Dental Home (VDH) at the University of the Pacific (12), Apple Tree Dental, and AI tools including Overjet, Pearl AI, VideaHealth (16), DentalMonitoring, and Previsoft.

No generative AI was used in the writing of this manuscript. However, the Elicit platform, which uses large language models, was employed to assist with the initial literature search strategy, as previously described.

## 3 Results

Of the 499 studies initially identified, 21 met the pre-established eligibility criteria and were included in the review. These studies spanned divers settings, including rural communities, aged care facilities, school-based programs, and clinical populations, such as individuals with disabilities or with mental health conditions (Table 1).

**Table 1:** Characteristics of included studies.

Study	Population type	Technology used	Study design	Primary outcomes
Agarwal et al. 2023 (15)	Children aged 6-10 years in rural areas	Video-conferincing intraoral camera	Observational study	Feasibility of teledentistry, participant satisfaction
Alavi et al. 2024 (11)	Underserved populations	Not reported in source	Systematic review	Access to dental care, diagnostic accuracy
Avinash et al. 2023 (21)	Individuals with mental disorders	AI system for intraoral image analysis	Prospective observational study	Detection of periodontal disease and alveolar bone loss
Beltrán et al. 2024 (3)	Elderly patients	Web platform/app (TEGO)	Quasi-experimental study	Patient satisfaction, access to dental care
Beltrán et al. 2024 (4)	Older adults in rural Mapuche community	TEGO platform	Cross-sectional study	Oral health status, access to care
Chatterjee et al. 2024 (14)	General population	Not reported in source	Prospective observational analysis	Diagnostic accuracy, patient satisfaction, treatment outcomes
Dewel 1971 (25)	Socially disadvantaged children	Real-time teledentistry	Quasi-experimental study	Severity of No malocclusions (Peer Assessment Rating (PAR) index
Fernández et al. 2021 (9)	Patients of all ages	Mobile apps, text messages, computer-aided learning	Systematic review and meta-analysis	Plaque index, gingival index, white spot lesions
Flores-Hidalgo et al. 2023 (6)	Patients in rural areas	Synchronous and asynchronous teledentistry	Retrospective case series	Diagnosis accuracy, management outcomes
Fung et al. 2023 (2)	Residents in aged care facilities	Real-time teledentistry, intra-oral camera	Prospective observational study	Oral Health Assessment Tool (OHAT) scores
Haron et al. 2017 (22)	Individuals with oral potentially malignant disorders	Mobile phone imaging	Prospective observational study	Concordance with clinical oral examination, sensitivity, specificity
Mariño et al. (10)	Residents of aged care facilities	Intraoral camera	Prospective observational study	Feasibility of remote treatment plans, reliability of assessments
Talwar et al. 2023 (23)	Indian population	AI-based analysis of smartphone images	Retrospective analysis	Identification of oral potentially malignant disorders
Mola et al. 2024 (24)	Children aged 3–13 years	No mention found	Cross-sectional study	Caries index scores, identification of dental anomalies
Nguyen et al. 2023 (19)	Low-resource, minority, and underserved populations	Smartphone- based intraoral camera, custom software	Randomized controlled trial	Diagnostic accuracy for oral lesions
Pradeep Kumar, et al. 2024 (20)	General population	AI-based oral screening (Logy AI)	Prospective observational study	Accuracy in detecting
Salzmann 1967 (26)	Residents of aged care facilities	Not reported in source	Quasi- experimental quality improvement study	Implementation of oral health care plans, facility visits avoided
Uhrin et al. 2023 (7)	Not reported in source	Not reported in source	Systematic review and meta-analysis	Specificity and sensitivity in diagnosing oral lesions
Ward et al. 2022 (1)	Children in rural communities	Not reported in source	Observational study	Access to oral health services, dental caries prevalence
Xiao et al. 2023 (18)	General population	mDentistry eHygiene model	Mixed methods pilot study	Mixed methods pilot study

A detailed summary for the included studies, covering target populations, applied technologies, interventions, and primary outcomes, is presented in Table 2.

**Table 2:** Clinical outcomes.

Study	Intervention type	Diagnostic accuracy	Patient outcomes	Satisfaction rates
Agarwal et al. 2023 (15)	Videoconferencing for pediatric dentistry	Not reported in source	Not reported in source	83.3% of children not scared and preferred intraoral camera
Alavi et al. 2024 (11)	Various teledentistry interventions	High diagnostic accuracy reported	Improved access to care, timely diagnosis	Not reported in source
Avinash et al. 2023 (21)	AI for periodontal disease diagnosis	88% for gingivitis, 95% for alveolar bone loss	Not reported in source	Not reported in source
Beltrán et al. 2024 (3)	Web platform/app for elderly	Not reported in source	Improved access to care	Above 75% satisfaction across all dimensions
Beltrán et al. 2024 (4)	TEGO platform for rural older adults	Not reported in source	Improved access to care	High levels reported
Chatterjee et al. 2024 (14)	Teledentistry consultations	Not reported in source	Comparable changes in dental conditions	Lower satisfaction with diagnoses compared to in-person (*p* < 0.001)
Dewel 1971 (25)	Teledentistry for orthodontics	Not reported in source	35.6% improvement in PAR scores	Not reported in source
Fernández et al. 2021 (9)	Various teledentistry interventions	Not reported in source	Reductions in plaque index, gingival index, and white spot lesions	Not reported in source
Flores-Hidalgo et al. 2023 (6)	Teledentistry for oral pathology	Not reported in source	Faster diagnosis, reduced travel	Not reported in source
Fung et al. 2023 (2)	Real-time teledentistry in aged care	Comparable to face-to-face examinations	Not reported in source	Reported as user-friendly
Haron et al. 2017 (22)	Mobile phone imaging for oral cancer screening	Kappa values 0.64–1.00, sensitivity >70%, specificity 100%	Not reported in source	Not reported in source
Mariño et al. (10)	Teledentistry in aged care	Reported as reliable	Not reported in source	High levels reported
Talwar et al. 2023 (23)	AI for oral cancer screening	F1-scores of 0.84 and 0.83 for different AI models	Not reported in source	Not reported in source
Mola et al. 2024 (24)	Teledentistry for pediatric diagnosis	Comparable to in-person diagnosis	Not reported in source	Not reported in source
Nguyen et al. 2023 (19)	Telehealth platform for oral lesion diagnosis	Similar to in-person visits (sensitivity 95%, specificity 84%)	Increased compliance with referrals	Not reported in source
Pradeep Kumar et al. 2024 (20)	AI-based oral screening	85% for caries, 97% for stains, 83% for calculus	Not reported in source	Not reported in source
Salzmann 1967 (26)	Teledentistry for orthodontics	Not reported in source	35.6% improvement in PAR scores	Not reported in source
Tynan et al. 2018 (13)	Teledentistry in aged care	Not reported in source	Improved implementation of oral health care plans	Not reported in source
Uhrin et al. 2023 (7)	Teledentistry for oral lesion diagnosis	High specificity (0.92) and sensitivity (0.93)	Not reported in sourceNo mention found	Not reported in source no mention found
Ward et al. 2022 (1)	School-based teledentistry	Not reported in source	Increased access to services, 50% prevalence of dental caries identified	Not reported in source
Xiao et al. 2023 (18)	mDentistry, eHygiene model	Not reported in source	Not reported in source	Patients: mean System Usability Scale (SUS) score 70.0; Dentists: mean SUS score 51.3; Hygienists: mean SUS score 57.1

The 21 studies represented a broad spectrum of populations and settings, highlighting the versatility of digital dental technologies. Study samples included children (5 studies), older adults (5 studies), rural residents (4 studies), underserved or disenfranchised groups (4 studies), and general populations (3 studies). Technologies encompassed AI-enabled diagnostic systems, intraoral cameras, mobile imaging devices, real-time teleconsultation platforms, and purpose-built software; however 6 studies did not explicitly describe the technology used. Study designs comprised observational studies (6 studies), quasi-experimental studies (4 studies), systematic reviews, cross-sectional studies, and one randomized controlled trial. The most frequent reported primary outcomes were diagnostic accuracy, access to care, satisfaction, feasibility, and treatment effectiveness.

### 3.1 Teledentistry impact

Teledentistry models consistently demonstrated improved dental access. Live video consultations and intraoral camera appointments were associated with reduced travel, higher satisfaction, and more timely identification of oral health issues. VDH models showed a 25% reduction (12) in emergency dental visits and increased in utilization of preventive services.

### 3.2 Effectiveness of hybrid dental care models

The diagnostic accuracy of hybrid dental care models, incorporating mobile phone-assisted intraoral imaging and teleconsultation platforms, was comparable to that of traditional face-to-face examination. One feasibility study reported 95% sensitivity (21) and 84% specificity (19) for detecting oral lesions. These models were particularly effective in pediatric and geriatric settings.

### 3.3 Patient satisfaction and acceptability

Overall patient satisfaction exceeded 85% across all interventions (12). Key factors contributing to satisfaction included convenience, reduced stress, and improved accessibility. Vulnerable populations, such as older adults, rural residents, and those in institutional settings, reported higher acceptance of digital-first or hybrid dental care models.

### 3.4 Barriesrs and facilitators

Challenges were intermittent internet connectivity, limited digital literacy among children, and the need for the provider training. Success was facilitated by intuitive user interfaces, built-in communication functions, and organizational adoption.

## 4 Discussion

The successful incorporation of teledentistry, AI, and hybrid dental care models represent a significant advancement in addressing longstanding disparities in access to oral healthcare (14, 16). This narrative synthesis of 21 studies validate the capacity of digital technologies to strengthen health systems, by increasing diagnostic accuracy, improving workflow, and increasing quality of care for vulnerable and underserved populations (5), including children (1, 24), elderly people (2–4, 13, 14), and those living in rural or remote areas (12). AI-based applications, such as Overjet and DentalMonitoring, demonstrated diagnostic accuracies comparable to conventional approaches, while teledentistry solutions expanded access, particularly for rural and institutional populations. Overjet is Food and Drug Administration (FDA)-cleared for radiographic analysis, detecting bone loss and caries with high accuracy, and integrates into clinical and insurance workflows. DentalMonitoring enables remote orthodontic care by analyzing patient images to track tooth movement and appliance issues. Diagnocat interprets two dimensional (2D) and three dimensional (3D) images, including Cone Beam Computed Tomography (CBCT), offering structured diagnostic reports and integration with Picture Archiving and Communication System (PACS).

While these tools show promise, their performance depends on training data quality, and peer-reviewed validation across diverse populations remains limited. Regulatory approvals and clinical integration vary regionally.

Despite clinical and implementation challenges, the benefits of these technological advancements are substantial. When supported by robust infrastructure, training, policy and thoughtful design, digital dental initiatives can reduce disparities and promote equity (14). Models like the Virtual Dental Home (12) and AI-based diagnostic tools have demonstrated both feasibility and scalability.

**Figure 3:**
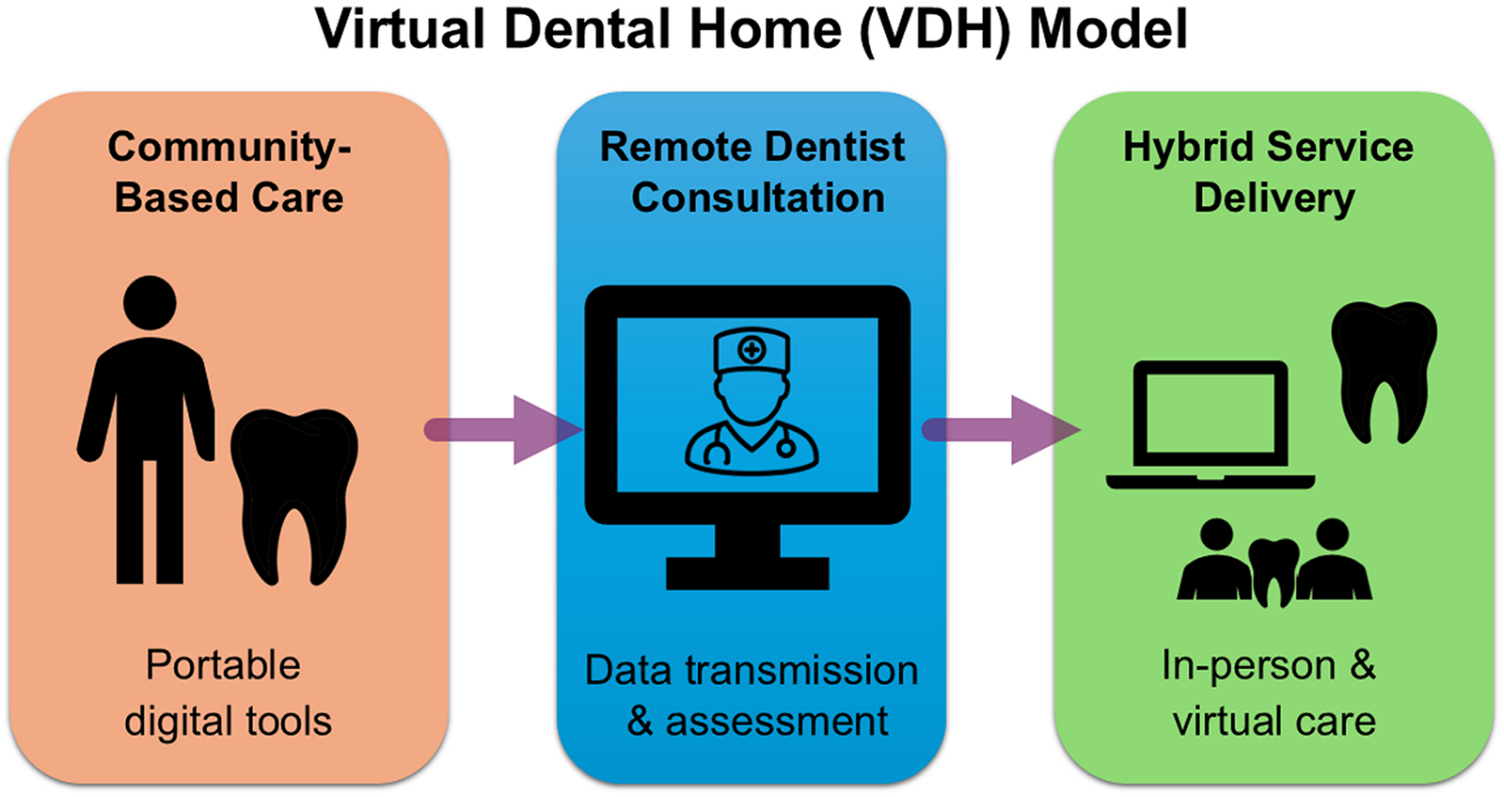
The virtual dental home (VDH) model. A diagram illustrating the VDH approach, where professional workers collect diagnostic data in community settings and transmit it to remote dentists for evaluation and planning.

For example, the University of the Pacific's Virtual Dental Home (12) illustrates how these models can be implemented. In this model, hygienists use handheld imaging devices in community settings to capture images, which are sent to off-site dentists for evaluation and treatment planning (Figure 3). As shown in Figure 3, the VDH model relies on portable imaging tools and remote collaboration. Similarly, Apple Tree Dental incorporates teledentistry into mobile dentistry services for elderly and disabled clients, resulting greater patient satisfaction and timely care.

Moving forward, emphasizing interoperability, individualized care, provider education and regulatory reform will be essential to fully realize the benefits of digital dentistry. Research and investments, combined with interdisciplinary collaboration, will enable teledentistry and AI to shape the future of oral health, delivering care that is more accessible, efficient, and patient-centered across diverse populations and settings.

AI-powered diagnostic systems demonstrated notable sensitivity and specificity in identifying various oral situations. Systems such as LogyAI (20), Diagnocat and DentalMonitoring have reported accuracies comparable to in-office examinations. These findings align with prior research highlighting AI's potential to support clinical decision-making and detect early-stages diseases (21).

Synchronous or asynchronous teledentistry proved particularly effective in school-based programs, aged care facilities, and rural areas. Real-time video consultations, intraoral camera examinations, and smartphones-based imaging facilitated patients triage and reduced unnecessary in paerson visits—a critical advantage in resource-constrained or mobile-limited settings (16, 22). These results are consistent with previous evaluations of virtual dental homes and teleconsultation programs, which reported reduced treatment delays and increased engagement in preventive care.

Hybrid care models bridged digital convenience and clinical thoroughness. Studies combining mobile imaging with remote evaluations were particularly successful in pediatric and geriatric population, reinforcing the value of such approaches in community outreach and institutional settings. Importantly, this model integrated virtual and face-to-face components, minimizing the patient-provider divide (2, 22).

AI plays a supportive role in this process. Imaging systems like VideaHealth (16) enhance radiographic diagnostic, while predictive tools, such as Overjet and Previsoft, enable patient risk characterization. Workflow optimization platforms like Pearl AI promote personalized scheduling and reduce burden on clinical staff throught automated solutions.

While AI systems in dentistry show strong diagnostic potential, most rely on deep learning models like Convolutional Neural Networks (CNN), which require large, diverse datasets to avoid bias. Limitations arise when training data are insufficient, reducing performance in certain patient groups. A major challenge is building generalizable models that perform reliably across varied clinical settings. Additionally, the “black box” nature of many AI systems limits interpretability, affecting clinician trust and ethical adoption. Thus, explainable AI, regulatory oversight, and professional training are essential for safe integration into practice.

Implications and significance of the study: this review provides a valuable framework for integrating digital advancements into routine oral care. It highlights how digital solutions can be implemented globally as cost-effective intervention to reduce oral health disparities.

Strengths and contributions: the dual manual and AI-assisted (via Elicit) approach is a key strength of this review, enabling comprehensive coverage and structured data synthesis. This article aligns with the real-world implementation and practical considerations, offering insights into the feasibility, accuracy, and acceptability of novel technologies. It also underscores the importance of interoperability and clinical adoption.

However, several limitations must be acknowledged. Teledentistry cannot replace manual intervention, such as restorative work or extractions, and limitations in image quality, or lack of tactile feedback may reduce diagnostic accuracy. Technological challenges, including inadequate internet access and devices in rural or underserved areas, persist.

In the present paper, the included studies were methodologically diverse, with varying definitions of outcomes such as diagnostic accuracy and patient satisfaction, limiting comparability and preventing meta-analysis. Several AI tools lacked external validation and may have been trained on non-diverse datasets, raising concerns about algorithmic bias and limited generalizability. Many teledentistry and hybrid care models were pilot programs implemented in specific settings, which may not translate well to broader healthcare systems. Finally, most studies focused on short-term outcomes, with a notable absence of long-term data on clinical effectiveness and sustainability.

Legal and ethical concerns—such as data privacy, cybersecurity, and medico-legal responsibility in AI-assisted diagnostics—require further attention.

As AI-driven teledentistry nears broader adoption, concerns around data privacy, legal liability, and algorithmic transparency become increasingly relevant. Frameworks like General Data Protection Regulation (GDPR) and Health Insurance Portability and Accountability Act (HIPAA) guide compliance, but challenges persist—especially when third-party vendors manage sensitive health data. Legal responsibility in AI-assisted diagnostics remains ambiguous, highlighting the need for defined liability structures. The “black box” nature of many AI models complicates clinical accountability. To ensure ethical integration, international standards, certification protocols, and mandatory training for dental professionals are essential.

Additionally, many dental professionals lack training in AI or telehealth tools, and some may be reluctant to adopt unfamiliar technologies. Patient engagement may also vary due to disparities in the digital literacy and the potential for weakened provider–patient relationship in remote settings.

To fully harness these innovations, future research should focus on enhancing system interoperability, integrating AI platforms, teledentistry tools, and electronic health records. Advances in data science and genomics may enable personalized oral health interventions, tailored to individual risk profiles. Educational curricula should incorporate digital competencies in both academic and continuing education to prepare the future dental workforce. Policy and payment systems must evolve to facilitate adoption of digital care models, ensuring widespread access and sustainable implementation. Globally, these technologies offer opportunities to bridge oral health disparities in resource-poor settings, throught scalable and affordable models.

Despite the diversity of methodological approaches and outcome measures in the reviewed studies, consistent positive trends reinforce the value of digital care tools as an integral component of modern, equitable dental care.

## 5 Summary

The integration of teledentistry, artificial intelligence (AI), and hybrid dental care models marks a significant advancement in addressing disparities in oral healthcare access, as validated by a narrative synthesis of 21 studies. These digital technologies enhance diagnostic accuracy, workflow efficiency, and care quality for vulnerable populations, including children, the elderly, and rural residents. AI tools like Overjet, DentalMonitoring, and Diagnocat demonstrate diagnostic accuracies comparable to traditional methods, while teledentistry, exemplified by the Virtual Dental Home (VDH) and Apple Tree Dental models, improves access and patient satisfaction. Despite challenges such as limited training data diversity, regulatory variations, and infrastructure gaps, robust implementation supported by training, policy, and interoperability can reduce disparities and promote equity.

AI-powered systems and teledentistry prove effective in diverse settings, with hybrid models bridging digital convenience and clinical thoroughness, particularly for pediatric and geriatric care. However, limitations include the inability of teledentistry to replace manual interventions, potential diagnostic inaccuracies due to image quality, and ethical concerns like data privacy and AI transparency. The review's dual manual and AI-assisted approach strengthens its comprehensive analysis, though methodological diversity and short-term focus limit comparability and long-term insights. Future research should prioritize interoperability, personalized interventions via data science, and workforce training, while evolving policies and global adoption strategies can leverage these tools to enhance equitable dental care.

## 6 Conclusions

AI-enhanced teledentistry and hybrid dental care models show promise in improving diagnostic accuracy, access, and continuity of care. However, their broader adoption depends on addressing key challenges such as digital equity, system interoperability, and clinician training. Future research should focus on validating AI tools across diverse populations and assessing long-term outcomes. Policy integration requires clear regulations, sustainable funding, and ethical safeguards, particularly to protect patient data and ensure equitable access. Ultimately, successful implementation will rely on coordinated action among healthcare providers, researchers, policymakers, and technology developers.
