# Correction: Innovations on the horizon: teledentistry, artificial intelligence, and hybrid models to improve oral health for vulnerable communities

**DOI:** 10.3389/froh.2025.1712116

**Published:** 2025-10-15

**Authors:** Sergiu Drafta, Andrei Macris, Alexandru E. Petre

**Affiliations:** Prosthetic Department, Faculty of Stomatology, “Carol Davila” University of Medicine and Pharmacy from Bucharest, Bucharest, Romania

**Keywords:** teledentistry, artificial intelligence, dental care, oral health, dental diagnostic

In the published article, there was no Acknowledgements statement. The authors affiliation to University of Medicine and Pharmacy “Carol Davila” from Bucharest offer us the access to the University program “Publish not Perish” (https://new.umfcd.ro/cercetare-si-dezvoltare/programe-de-sprijinire-a-cercetarii/programul-publish-notperish/). Through this program the authors could be reimbursed the publication fee, according to the program conditions.

The mentioned program has been slightly changed among the period of time from the beginning of our manuscript submission and present day, without a prior announcement, conditioning us for mention to the Acknowledgment section the following: “Publication of this paper was supported by the University of Medicine and Pharmacy Carol Davila, through the institutional program Publish not Perish” (chrome-extension://efaidnbmnnnibpcajpcglclefindmkaj/https://umfcd.ro/wp-content/uploads/2025/CERCETARE_SI_DEZVOLTARE/PROGRAMUL_PUBLISH_NOT_PERISH/METODOLOGIA%20PROGRAM%20PUBLISH%20NOT%20PERISH%2BANEXE%20aprobat%20CA%2BSenat.pdf).

This mention is referring out just for the publication fee, therefore our previous statement that our study received no fund still stands out. All authors are agreeing with this decision.

Acknowledgement correction:

“Publication of this paper was supported by the University of Medicine and Pharmacy Carol Davila, through the institutional program Publish not Perish”.

